# Supplementary material for: Non-visual photoreceptive brain specification in sea urchin larvae
Source: Nat Commun. 2025 Nov 19;16:10054. doi: 10.1038/s41467-025-65628-9 (PMC12630818; doi:10.1038/s41467-025-65628-9)
Supplement: Supplementary file 1 — Supplementary Information [file 41467_2025_65628_MOESM1_ESM.docx]

**Non-Visual Photoreceptive Brain Specification in Sea Urchin Larvae**

^†^Junko Yaguchi^1^, *^†^Koki Tsuyuzaki^2,3,4,5^, Kazumi Sakai^6^, Naoaki Sakamoto^7^, Takashi Yamamoto^7^, Takahiro Yamashita^6^, *Shunsuke Yaguchi^1,5^

*Corresponding authors; Shunsuke Yaguchi, Shimoda Marine Research Center, University of Tsukuba, 5-10-1 Shimoda, Shizuoka, 415-0025 Japan

Phone; +81-558-22-1317

Fax; +81-558-22-0346

E-mail; yag@shimoda.tsukuba.ac.jp

Koki Tsuyuzaki, Department of Artificial Intelligence Medicine, Graduate School of Medicine, Chiba University, Chiba 260-8670, Japan

Phone; +81-43-222-7171

E-mail; koki.tsuyuzaki@gmail.com

**Supplementary Figures**


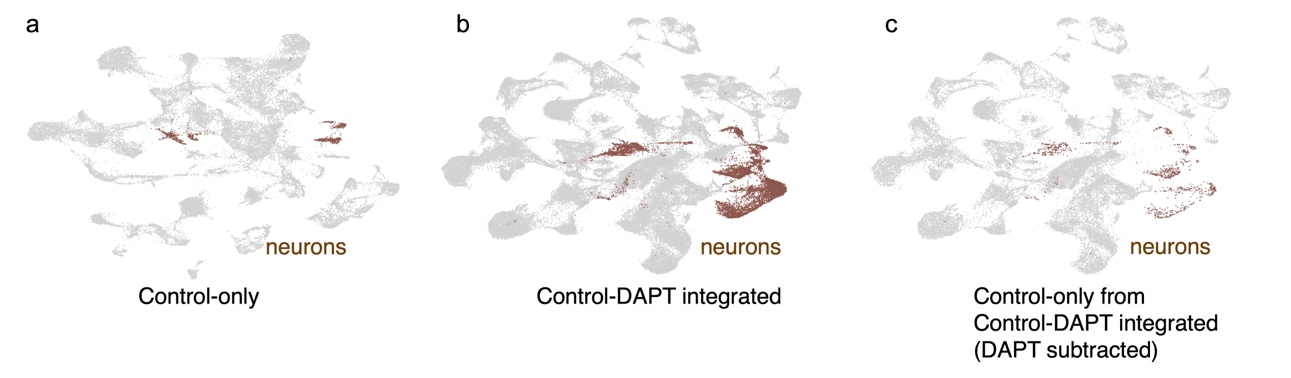


**Supplementary Figure 1. Comparison of Control-only UMAP (a) vs. control-DAPT integrated UMAP (b) vs. control-only from Control-DAPT integrated (DAPT-subtracted) UMAP (c).** The control-DAPT integrated UMAP (b), and the subsequent control-only UMAP (generated by subtracting DAPT from the integrated UMAP) (c), revealed a finer subdivision of neural regions compared to the control-only UMAP (a). All UMAPs presented in this paper represent control-only data derived from the control-DAPT integrated dataset, with DAPT-subtracted.


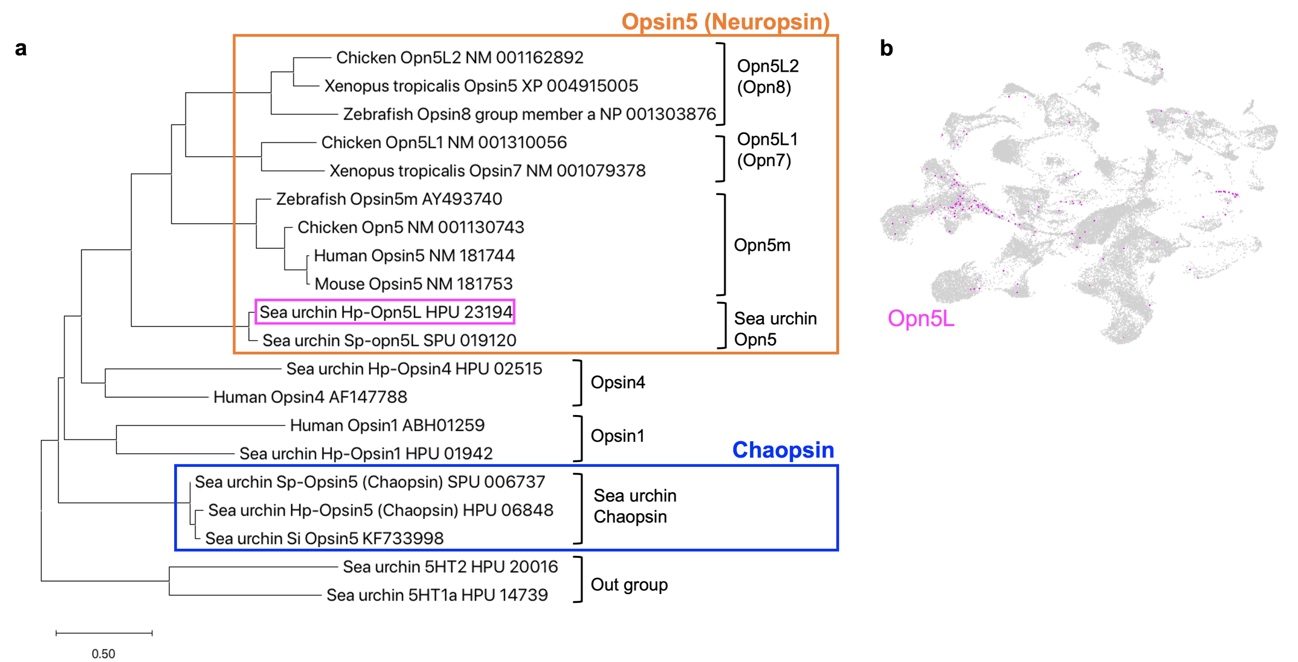


**Supplementary Figure 2. Phylogenetic classification and expression pattern of *opn5L* in UMAP.** **a.** Phylogenetic tree of opsin 5 and other opsin genes, constructed using the motif sequence of the G-protein coupled receptor family 1 with the maximum-likelihood method in MEGA11 software. The target of this study, Hp-Opn5L(highlighted in magenta) from the sea urchin, belongs to the Neuropsin group (orange rectangle), with homologous genes present in many deuterostomes. In contrast, Sp-Opsin5, a relatively well-studied opsin in sea urchins, belongs to the Chaopsin group (blue rectangle), which is phylogenetically distinct from the Opsin5 (Neuropsin) group found in mammals. b. *opn5L* expression pattern in UMAP.


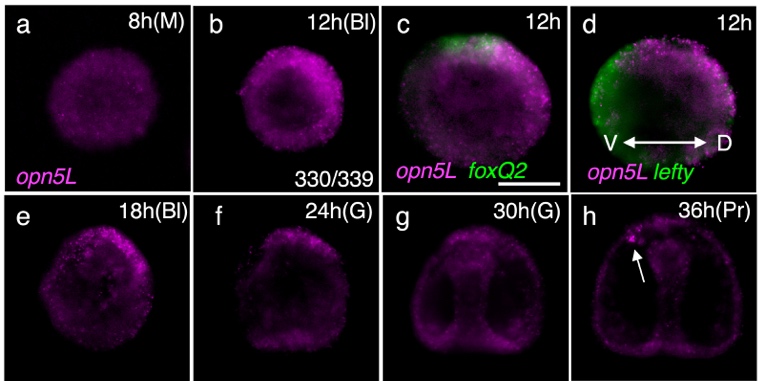


**Supplementary Figure 3. Developmental expression pattern of *opn5L* in sea urchin larvae.** **a–l.** Expression pattern of *opn5L* from 8-hour to 72-hour larvae. Expression of *opn5L* was first detected at 12 hours (**b**). While *opn5L* expression partially overlapped with *foxQ2*, it was strongly biased to one side (**c**). This dorsal bias in *opn5L* expression was further confirmed by double staining with *lefty*, a ventral marker. V, ventral; D, dorsal. Expression of *opn5L* gradually weakened from 24-hour larvae onward, but at 36 hours, it was still detectable around brain region at the cellular level. M, morula; Bl, blastula; G, gastrula; Pr, prism.


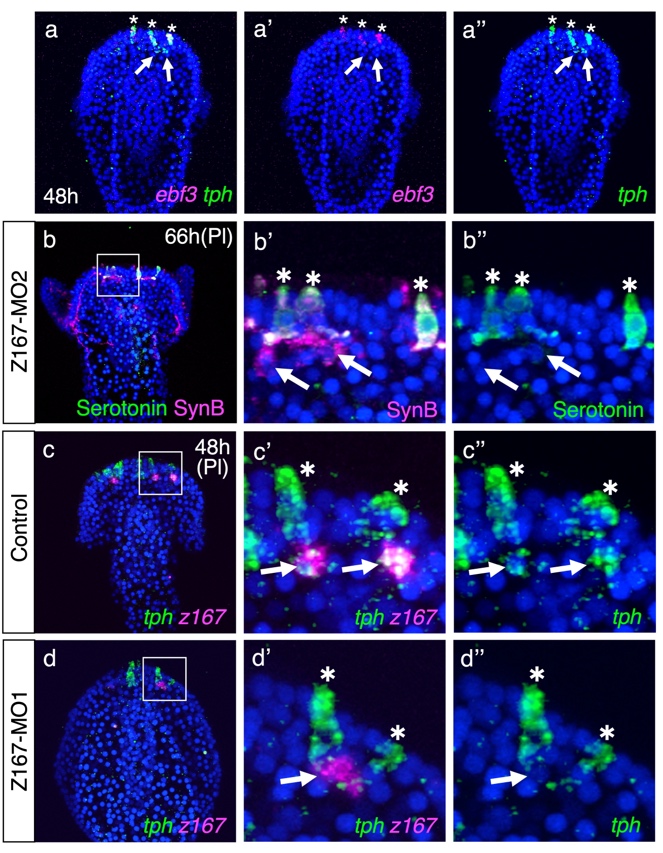


**Supplementary Figure 4. Loss of dorsal serotonergic neurons in z167 morphants. a.** Expression pattern of *ebf3* and *tph* at 48 h. *ebf3* is exclusively expressed in the anterior serotonergic neurons (**a’**), not in the dorsal serotonergic neurons (**a’’**; arrows). **b.** Expression pattern of serotonin and SynaptotagminB (SynB) in z167-MO2-injected larvae. In z167-MO2 morphants, serotonin was absent in dorsal serotonergic neurons (arrows), consistent with the phenotype observed in z167-MO1 morphants in Fig. 2**v**. The boxed region in (**b**) is magnified in (**b’** and **b’’**). **c, d.** Expression pattern of *tph* and *z167* in control (**c**) and z167-MO1-injected larvae (**d**). In z167-MO1 morphants, *z167* expression was detected, but *tph* was absent in dorsal serotonergic neurons (arrows). Asterisks indicate anterior serotonergic neurons. The boxed regions in (**c, d**) are magnified in (**c’, c’’**) and (**d’, d’’**), respectively.


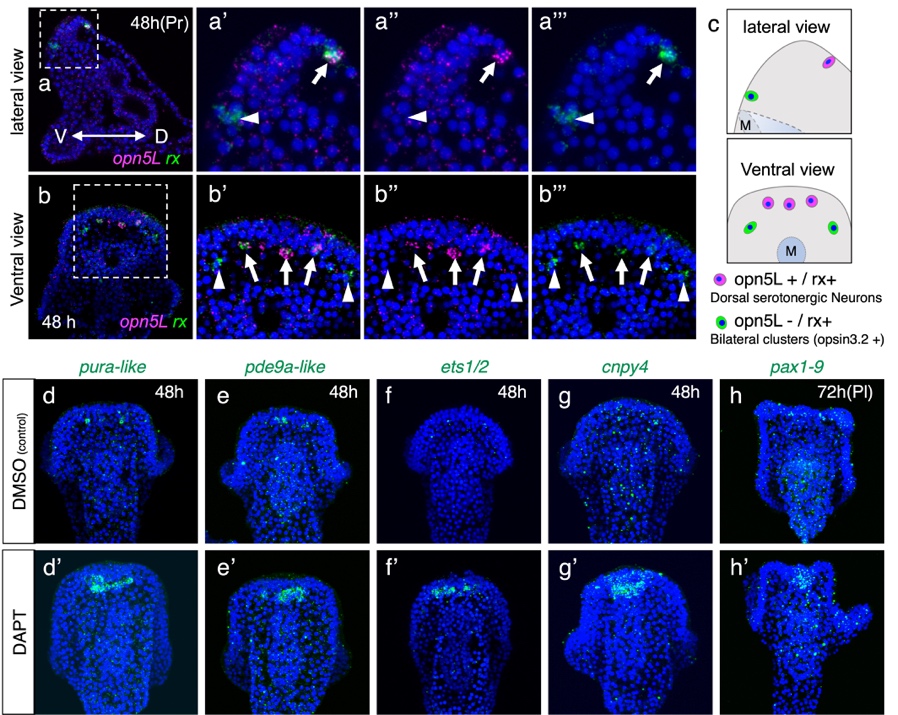


**Supplementary Figure 5. Co-expression patterns of opn5L and rx, and gene expressions in dorsal serotonergic neurons. a, b.** Co-expression pattern of *opn5L* and *rx*, shown in lateral and ventral views. *rx* and *opn5L* were co-expressed in dorsal serotonergic neurons (arrows in **a’–a’’’** and **b’–b’’’**). *rx* was also detected in the left and right ventral anterior regions, where *opn3.2* is expressed (arrowheads in **a’–a’’’** and **b’–b’’’**); however, *opn5L* was not expressed in these cells. The boxed regions in (**a, b**) are magnified in (**a’–a’’’**) and (**b’–b’’’**), respectively. V, ventral; D, dorsal. **c.** Schematic representation of (**a, b**). M, mouth. **d–h.** Expression patterns of *pura-like*, *pde9a-like*, *ets1/2*, *cnpy4*, and *pax1-9* in 48- or 72-hour larvae treated with DMSO (control) (**d–h**) and DAPT (**d’–h’**). These genes exhibited higher expression levels in dorsal serotonergic neurons, consistent with the single-cell analysis results shown in Fig. 2**a–h.**


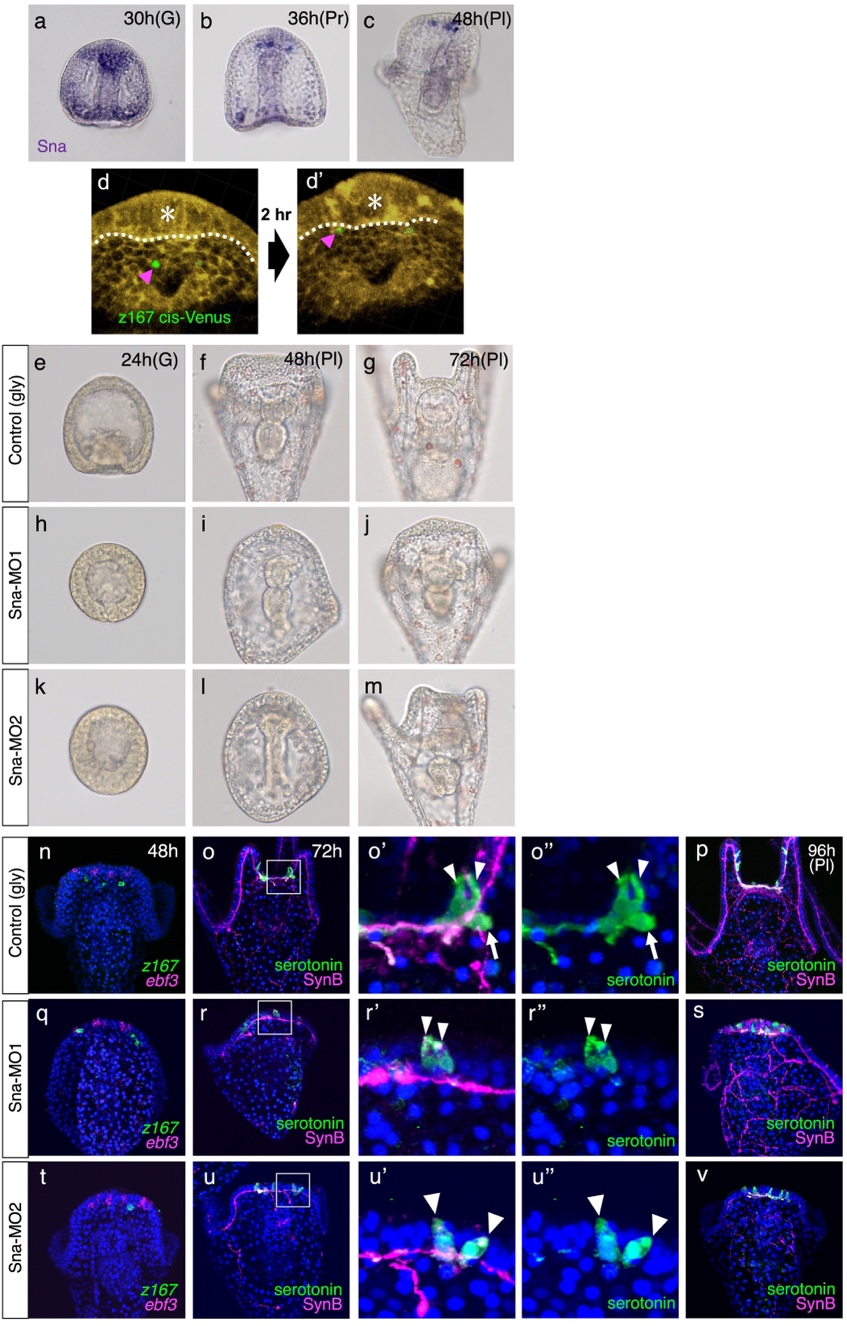


**Supplementary Figure 6. Sna expression and its role in sea urchin development. a–c.** Expression pattern of *sna*, detected using the alkaline phosphatase (AP) chromogenic system. **d.** Frames from Movie 1, showing a 50-hour larva microinjected with z167 cis-Venus. In these embryos, fluorescent cells were observed in the anterior ectoderm (17.2%), other ectodermal regions (2.0%), and the mesoderm (2.2%) (n = 402). The left panel shows the initial frame, and the right panel shows the final frame after two-hour recording. Fluorescent cells initially located away from the region containing anterior serotonergic neurons (*) (**d**) migrated toward the area during the recording period (**d’**). The arrowhead indicates a fluorescent cell that moved to the region containing anterior serotonergic neurons (*). **e–m.** Transmission images of control, Sna-MO1, and Sna-MO2-injected larvae. **n, q, t.** *z167* and *ebf3* expression pattern in control larva (m), Sna-MO1 morphant (q), and Sna-MO2 morphant (t). The average numbers of cells expressing *ebf3* per larvae were 3.96 ± 0.09 in control, 3.18 ± 0.36 in Sna-MO1 morphants, and 3.45 ± 0.11 in Sna-MO2 morphants. The average numbers of cells expressing *z167* per larvae were 2.64 ± 0.06 in control, 0.98 ± 0.41 in Sna-MO1 morphants, and 1.96 ± 0.5 in Sna-MO2 morphants (N = 4, control; n = 11, 38, 23, 16, Sna-MO1; n = 18, 22, 41, 64, Sna-MO2; n = 16, 20, 27, 44). **o, p, r, s, u, v.** Serotonin and synB expression pattern in control larva (o, p), Sna-MO1 morphant (r, s), and Sna-MO2 morphant (u, v). The rate of dorsal serotonergic neurons existence was 100 % (n = 40) in control, 28.1 % (n = 32) in Sna-MO1 morphants and 82.1% (n = 28) in Sna-MO2 morphants at 72-hour.


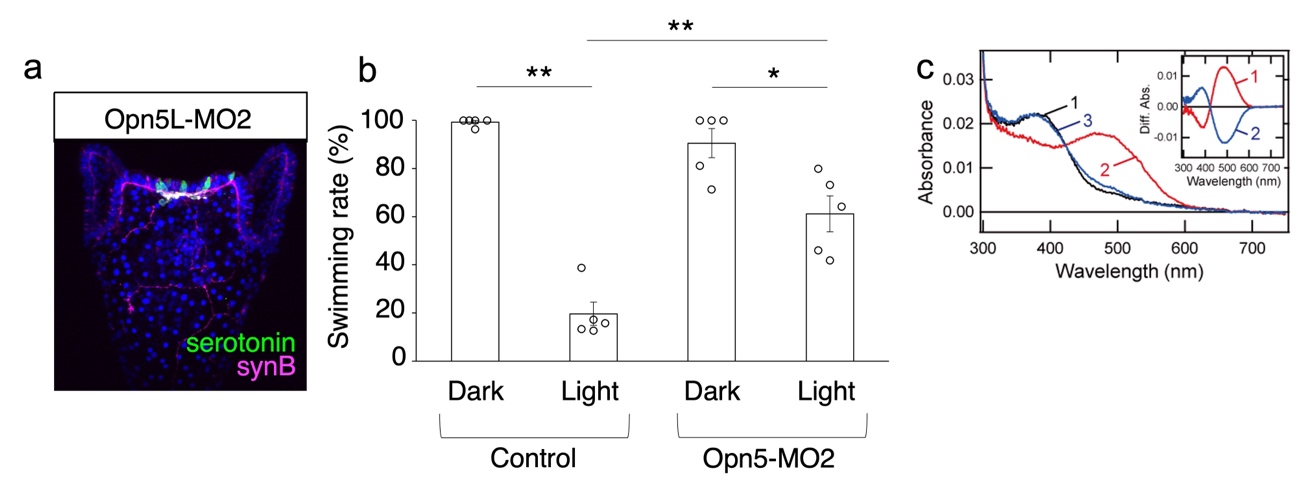


**Supplementary Figure 7. Opn5L functions in regulating swimming behavior in response to light. a.** Opn5L-MO2 moprhants exibit normal morphology and neural patterns. **b.** Opn5L-MO2 morphants showed slightly weaker effects than Opn5L-MO1 after prolonged light exposure, but sinking behavior was still reduced. Photon flux density was set to 150–200 μmol m⁻² s⁻¹. The mean swimming rate in control larvae was 99.3% ± 0.7% SEM (n = 28, 12, 45, 24, 38) in the dark and 19.7% ± 4.9% SEM (n =19, 39, 52, 23, 18) in the light. Opn5-MO2-injected larvae showed a mean swimming rate of 90.5% ± 6.0% SEM (n = 20, 7, 74, 12, 32) in the dark and 61.2% ± 7.4% SEM (n = 14, 15, 50, 13, 10) in the light. ﻿To compare the two groups, ﻿we used Welch’s t-test (two-tailed) with a significance level of 0.001,0.01or 0.05. **, *p*<0.01; *, *p*<0.5; n.s., not significant. **c.** Absorption spectra of *S. purpuratus* Opn5L protein. Recombinant proteins of *S. purpuratus* Opn5L were purified after the addition of 11-*cis* retinal.  Absorption spectra were recorded in the dark (curve 1), after UV light (360 nm) irradiation (curve 2) and after subsequent yellow light (>500 nm) irradiation (curve 3).  The spectrum of purified Opn5L protein had a peak at around 390 nm.  UV light irradiation shifted the spectrum from the UV region to the visible region (~500 nm) and subsequent yellow light irradiation recovered the absorbance in the UV region.  (inset) Spectral changes of *S. purpuratus* Opn5 caused by UV light irradiation (curve 1) and subsequent yellow light irradiation (curve 2).


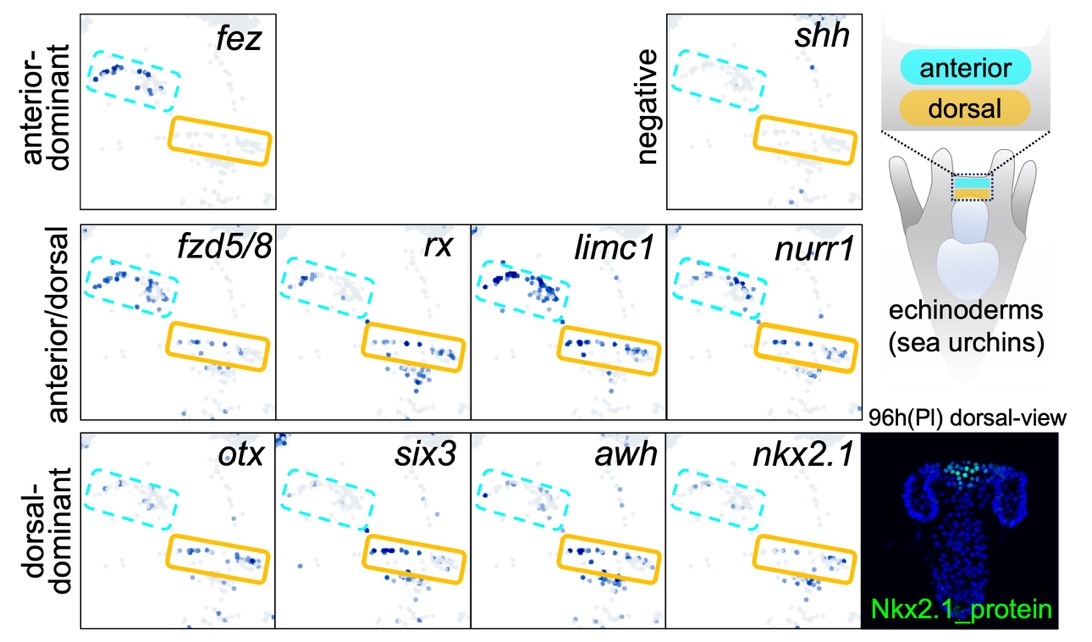


**Supplementary Figure 8. Gene expression profile of sea urchin pre-oral brain-like region in UMAP.** *fez* is clearly expressed dominantly in anterior serotonergic neuron cluster. *fzd5/8*, *rx*, *limc1* (*lhx2/9*), and *nurr1* are expressed in both clusters. *otx*, *six3*, *awh* (*lhx6*), and *nkx2.1*are dominantly expressed in dorsal serotonergic neurons, which express *opn5L*. Immunostaining for Nkx2.1 revealed its expression in the dorsal serotonergic neuron domain. *shh* is not expressed in serotonergic neurons.
