# Supplementary material for: Non-visual photoreceptive brain specification in sea urchin larvae
Source: Nat Commun. 2025 Nov 19;16:10054. doi: 10.1038/s41467-025-65628-9 (PMC12630818; doi:10.1038/s41467-025-65628-9)
Supplement: Supplementary file 3 — Description of Additional Supplementary Files [file 41467_2025_65628_MOESM3_ESM.pdf]

Description of Additional Supplementary Files

File Name: Supplementary Movie 1
Description: **Z167 cell migration**
Cells visualized with the Z167 promoter represent precursors of dorsal serotonergic neurons and, within approximately two hours, shift their position toward the anterior serotonergic neurons. Dashed line: boundary of anterior serotonergic neurons. Magenta: Z167 expression.
