# Supplementary material for: Non-visual photoreceptive brain specification in sea urchin larvae
Source: Nat Commun. 2025 Nov 19;16:10054. doi: 10.1038/s41467-025-65628-9 (PMC12630818; doi:10.1038/s41467-025-65628-9)
Supplement: Supplementary file 5 — Reporting Summary [file 41467_2025_65628_MOESM5_ESM.pdf]

nature portfolio

Corresponding author(s): Shunsuke Yaguchi

Last updated by author(s): Sep 26, 2025

Reporting Summary

Nature Portfolio wishes to improve the reproducibility of the work that we publish. This form provides structure for consistency and transparency in reporting. For further information on Nature Portfolio policies, see our Editorial Policies and the Editorial Policy Checklist.

Statistics

For all statistical analyses, confirm that the following items are present in the figure legend, table legend, main text, or Methods section.

n/a	Confirmed	
☐	☒	The exact sample size (*n*) for each experimental group/condition, given as a discrete number and unit of measurement
☐	☒	A statement on whether measurements were taken from distinct samples or whether the same sample was measured repeatedly
☐	☒	The statistical test(s) used AND whether they are one- or two-sided<br>*Only common tests should be described solely by name; describe more complex techniques in the Methods section.*
☐	☒	A description of all covariates tested
☐	☒	A description of any assumptions or corrections, such as tests of normality and adjustment for multiple comparisons
☐	☒	A full description of the statistical parameters including central tendency (e.g. means) or other basic estimates (e.g. regression coefficient) AND variation (e.g. standard deviation) or associated estimates of uncertainty (e.g. confidence intervals)
☐	☒	For null hypothesis testing, the test statistic (e.g. *F*, *t*, *r*) with confidence intervals, effect sizes, degrees of freedom and *P* value noted<br>*Give P values as exact values whenever suitable.*
☒	☐	For Bayesian analysis, information on the choice of priors and Markov chain Monte Carlo settings
☒	☐	For hierarchical and complex designs, identification of the appropriate level for tests and full reporting of outcomes
☒	☐	Estimates of effect sizes (e.g. Cohen's *d*, Pearson's *r*), indicating how they were calculated

Our web collection on statistics for biologists contains articles on many of the points above.

Software and code

Policy information about availability of computer code

Data collection	n/a
Data analysis	n/a

For manuscripts utilizing custom algorithms or software that are central to the research but not yet described in published literature, software must be made available to editors and reviewers. We strongly encourage code deposition in a community repository (e.g. GitHub). See the Nature Portfolio guidelines for submitting code & software for further information.

Data

Policy information about availability of data

All manuscripts must include a data availability statement. This statement should provide the following information, where applicable:

- Accession codes, unique identifiers, or web links for publicly available datasets
- A description of any restrictions on data availability
- For clinical datasets or third party data, please ensure that the statement adheres to our policy

Source data for all Figures and Supplementary Figures are provided with the paper. Sequence data can be found in the genome database of Hemicentrotus pulcherrimus, HpBase (http://cell-innovation.nig.ac.jp/Hpul/)76. Other data are available from the corresponding author upon request.
For Editors and Reviewers only (not public until the paper is published)
The raw and processed next generation sequencing data have been deposited in NCBI's Gene Expression Omnibus and are accessible through GEO Series accession number GSE265747. Secure Token for GEO: mzkleqswrpevrqr

Research involving human participants, their data, or biological material

Policy information about studies with human participants or human data. See also policy information about sex, gender (identity/presentation), and sexual orientation and race, ethnicity and racism.

Reporting on sex and gender	n/a
Reporting on race, ethnicity, or other socially relevant groupings	n/a
Population characteristics	n/a
Recruitment	n/a
Ethics oversight	n/a

Note that full information on the approval of the study protocol must also be provided in the manuscript.

Field-specific reporting

Please select the one below that is the best fit for your research. If you are not sure, read the appropriate sections before making your selection.

☐ Life sciences ☐ Behavioural & social sciences ☒ Ecological, evolutionary & environmental sciences

For a reference copy of the document with all sections, see nature.com/documents/nr-reporting-summary-flat.pdf

Ecological, evolutionary & environmental sciences study design

All studies must disclose on these points even when the disclosure is negative.

Study description	This study discusses brain evolution based on the findings from scRNA-seq analysis of sea urchin larvae, revealing that the anterior neuroectoderm of sea urchin larvae possesses a gene repertoire similar to that of the vertebrate forebrain.
Research sample	Sea urchins, Hemicentrotus pulcherrimus<br>Culture cells, HEK293
Sampling strategy	No sample-size calculations were performed. Sample size was determined to be adequate based on the magnitude and consistency of measurable differences between groups.
Data collection	All authors collected all data. The methods are described in Materials and Methods. The numbers of larvae were counted under microscopes, and the numbers of cells and the size of tissues were measured on computers.
Timing and spatial scale	The experiments have been performed since October 2019. There is no periodical gap since then.
Data exclusions	On principle, data were only excluded for bad growth rate batches (unhealthy egg and sperm combinations).
Reproducibility	Replicate experiments were successful.
Randomization	We used different pairs of male and female for each experiment because the sea urchins are collected from wild habitats.
Blinding	The data presented here did not require the use of blinding.

Did the study involve field work? ☐ Yes ☒ No

Reporting for specific materials, systems and methods

We require information from authors about some types of materials, experimental systems and methods used in many studies. Here, indicate whether each material, system or method listed is relevant to your study. If you are not sure if a list item applies to your research, read the appropriate section before selecting a response.

Materials & experimental systems

n/a	Involved in the study	
☐	☒	Antibodies
☐	☒	Eukaryotic cell lines
☒	☐	Palaeontology and archaeology
☐	☒	Animals and other organisms
☒	☐	Clinical data
☒	☐	Dual use research of concern
☒	☐	Plants

Methods

n/a	Involved in the study	
☒	☐	ChIP-seq
☒	☐	Flow cytometry
☒	☐	MRI-based neuroimaging

Antibodies

Antibodies used	mouse anti-Synaptotagmin (Nakajima et al., 2004 Evol Dev), rabbit anti-serotonin (#S5545, Sigma-Aldrich), goat anti-mouse IgG conjugated with Alexa 568 (Thermo Fischer), goat anti-rabbit IgG conjugated with Alexa 488 (Thermo Fischer), goat anti-mouse IgG conjugated with HRP (BioLegend), sheep anti-Digoxygenin antibody Fab fragment conjugated with peroxydase or alkaline phosphatase (Roche).
Validation	All antibodies used in this study were validated in the referred previous reports or by suppliers.

Eukaryotic cell lines

Policy information about cell lines and Sex and Gender in Research

Cell line source(s)	HEK293S cells were kindly provided from Prof. J. Nathans (Johns Hopkins School of Medicine, Baltimore, MD).
Authentication	HEK293S cells were characterized and were authenticated as described (Nathans et al., (1989) Vision Res. 29, 907).
Mycoplasma contamination	HEK293S cells were tested negative for mycoplasma contamination.
Commonly misidentified lines (See ICLAC register)	HEK293S cell line was not listed by ICLAC.

Animals and other research organisms

Policy information about studies involving animals; ARRIVE guidelines recommended for reporting animal research, and Sex and Gender in Research

Laboratory animals	n/a
Wild animals	Hemicentrotus pulcherrimus were collected by hands under the permission of Japan Fisheries Cooperatives and local governments around Shimoda Marine Research Center, University of Tsukuba, around the Marine and Coastal Research Center, Ochanomizu University and around Research Center for Marine Biology, Tohoku University. Wet macro algae or wet newspapers were used for their shipment. After the study, the sea urchins were kept in the overflowed tank with feeding three times a week as long as they are survived. When the next breeding season comes, they are used for new experiments.
Reporting on sex	n/a
Field-collected samples	Until use for the study, the sea urchins are kept in their breeding season temperature (16 ˚C) with enough food in the closed aquarums. After used, they are transferred to the overflowed tank. Photoperiods are same as outside. We do not intentionally terminate their lives for our study.
Ethics oversight	No ethical approval or guidance was required in our university for sea urchin studies because our experiments do not contain any genetic recombination methods. Animal collection was performed with appropriate prefectures and fishery cooparative permitting.

Note that full information on the approval of the study protocol must also be provided in the manuscript.

Plants

Seed stocks	*Report on the source of all seed stocks or other plant material used. If applicable, state the seed stock centre and catalogue number. If plant specimens were collected from the field, describe the collection location, date and sampling procedures.*
Novel plant genotypes	*Describe the methods by which all novel plant genotypes were produced. This includes those generated by transgenic approaches, gene editing, chemical/radiation-based mutagenesis and hybridization. For transgenic lines, describe the transformation method, the number of independent lines analyzed and the generation upon which experiments were performed. For gene-edited lines, describe the editor used, the endogenous sequence targeted for editing, the targeting guide RNA sequence (if applicable) and how the editor was applied.*
Authentication	*Describe any authentication procedures for each seed stock used or novel genotype generated. Describe any experiments used to assess the effect of a mutation and, where applicable, how potential secondary effects (e.g. second site T-DNA insertions, mosiacism, off-target gene editing) were examined.*
